# Supplementary material for: Surface Microbiome Profiling of Dental Elevators Using Third‐Generation Sequencing: Implications for Infection Control in Dental Practice
Source: Microbiologyopen. 2025 Dec 8;14(6):e70178. doi: 10.1002/mbo3.70178 (PMC12685768; doi:10.1002/mbo3.70178)
Supplement: Supplementary file 1 — customer_backup. [file MBO3-14-e70178-s001.zip › customer_backup/customer_backup/function_analysis/KEGG/allsample/K2species/allsample.reabundance_Class2Family.txt.pdf]

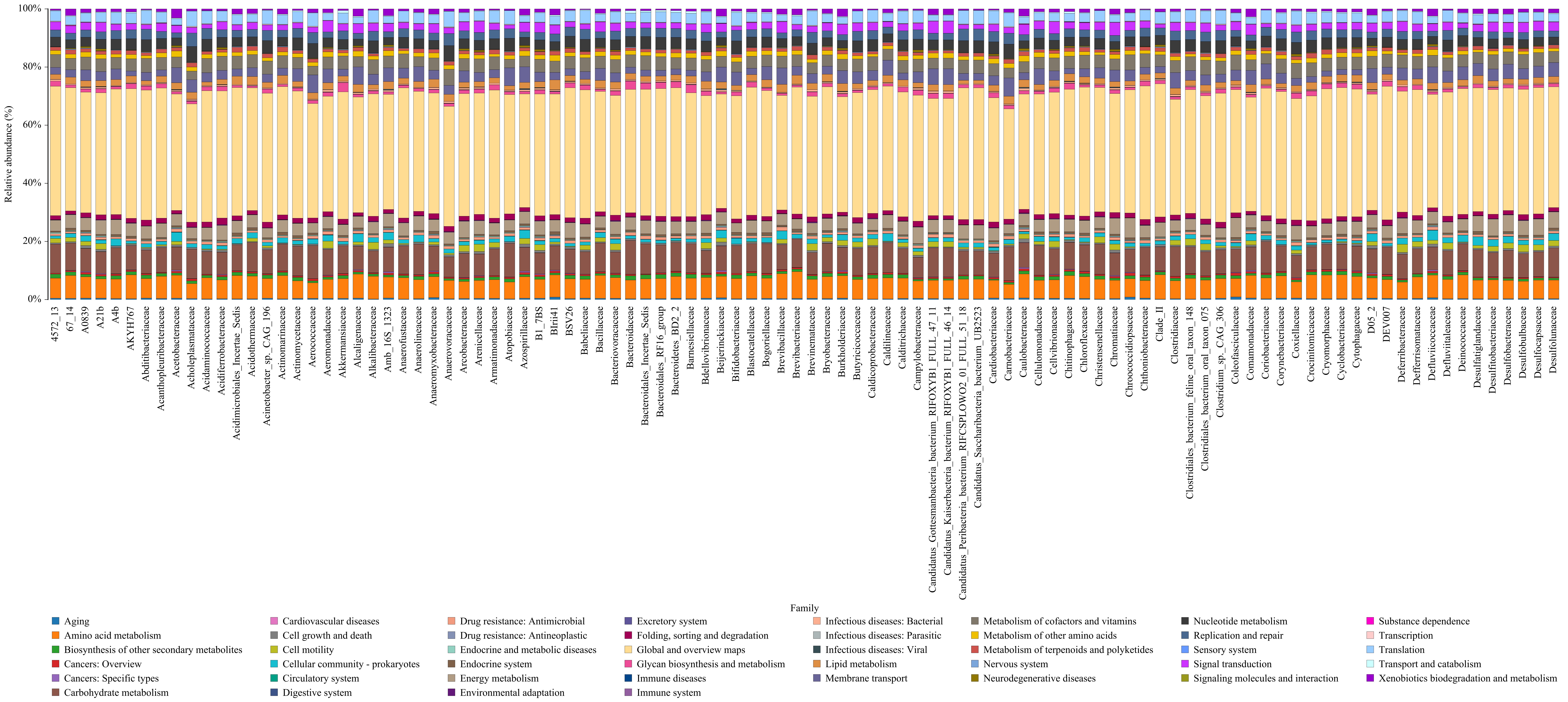
Relative abundance (%)
100%
80%
60%
40%
20%
0%
4572_13
67_14
A0839
A21b
A4b
AKYH767
Abditibacteriaceae
Acanthopleuribacteraceae
Acetobacteraceae
Acholeplasmataceae
Acidaminococcaceae
Acidiferrobacteraceae
Acidimicrobiales_Incertae_Sedis
Acidothermaceae
Acinetobacter_sp._CAG_196
Actinomarinaceae
Actinomycetaceae
Aerococcaceae
Aeromonadaceae
Akkermansiaceae
Alcaligenaceae
Alkalibacteraceae
Amb_16S_1323
Anaerofustaceae
Anaerolineaceae
Anaeromyxobacteraceae
Anaerovoracaceae
Arcobacteraceae
Arenicellaceae
Armatimonadaceae
Atopobiaceae
Azospirillaceae
B1_7BS
BIrii41
BSV26
Babeliaceae
Bacillaceae
Bacteriovoracaceae
Bacteroidaceae
Bacteroidales_Incertae_Sedis
Bacteroidales_RF16_group
Bacteroidetes_BD2_2
Barnesiellaceae
Bdellovibrionaceae
Beijerinckiaceae
Bifidobacteriaceae
Blastocatellaceae
Bogoriellaceae
Brevibacillaceae
Brevibacteriaceae
Brevinemataceae
Bryobacteraceae
Burkholderiaceae
Butyricicoccaceae
Caldicoprobacteraceae
Caldilineaceae
Calditrichaceae
Campylobacteraceae
Candidatus_Gottesmanbacteria_bacterium_RIFOXYB1_FULL_47_11
Candidatus_Kaiserbacteria_bacterium_RIFOXYB1_FULL_46_14
Candidatus_Peribacteria_bacterium_RIFCSPLOWO2_01_FULL_51_18
Candidatus_Saccharibacteria_bacterium_UB2523
Cardiobacteriaceae
Carnobacteriaceae
Caulobacteraceae
Cellulomonadaceae
Cellvibrionaceae
Chitinophagaceae
Chloroflexaceae
Christensenellaceae
Chromatiaceae
Chroococcidiopsaceae
Chthoniobacteraceae
Clade_II
Clostridiaceae
Clostridiales_bacterium_feline_oral_taxon_148
Clostridiales_bacterium_oral_taxon_075
Clostridium_sp._CAG_306
Coleofasciculaceae
Comamonadaceae
Coriobacteriaceae
Corynebacteriaceae
Coxiellaceae
Crocinitomicaceae
Cryomorphaceae
Cyclobacteriaceae
Cytophagaceae
D05_2
DEV007
Deferribacteraceae
Defferrisomataceae
Defluviicoccaceae
Defluviitaleaceae
Deinococcaceae
Desulfatiglandaceae
Desulfitobacteriaceae
Desulfobacteraceae
Desulfobulbaceae
Desulfocapsaceae
Desulfolunaceae
Family
Aging
Amino acid metabolism
Biosynthesis of other secondary metabolites
Cancers: Overview
Cancers: Specific types
Carbohydrate metabolism
Cardiovascular diseases
Cell growth and death
Cell motility
Cellular community - prokaryotes
Circulatory system
Digestive system
Drug resistance: Antimicrobial
Drug resistance: Antineoplastic
Endocrine and metabolic diseases
Endocrine system
Energy metabolism
Environmental adaptation
Excretory system
Folding, sorting and degradation
Global and overview maps
Glycan biosynthesis and metabolism
Immune diseases
Immune system
Infectious diseases: Bacterial
Infectious diseases: Parasitic
Infectious diseases: Viral
Lipid metabolism
Membrane transport
Metabolism of cofactors and vitamins
Metabolism of other amino acids
Metabolism of terpenoids and polyketides
Nervous system
Neurodegenerative diseases
Nucleotide metabolism
Replication and repair
Sensory system
Signal transduction
Signaling molecules and interaction
Substance dependence
Transcription
Translation
Transport and catabolism
Xenobiotics biodegradation and metabolism
